# Bayesian Modeling of Biomolecular Assemblies with Cryo-EM Maps

**DOI:** 10.3389/fmolb.2017.00015

**Published:** 2017-03-22

**Authors:** Michael Habeck

**Affiliations:** ^1^Statistical Inverse Problems in Biophysics, Max Planck Institute for Biophysical ChemistryGöttingen, Germany; ^2^Felix Bernstein Institute for Mathematical Statistics in the Biosciences, University of GöttingenGöttingen, Germany

**Keywords:** cryo-EM, modeling, Bayesian inference, Markov chain Monte Carlo, inferential structure determination

## Abstract

A growing array of experimental techniques allows us to characterize the three-dimensional structure of large biological assemblies at increasingly higher resolution. In addition to X-ray crystallography and nuclear magnetic resonance in solution, new structure determination methods such cryo-electron microscopy (cryo-EM), crosslinking/mass spectrometry and solid-state NMR have emerged. Often it is not sufficient to use a single experimental method, but complementary data need to be collected by using multiple techniques. The integration of all datasets can only be achieved by computational means. This article describes Inferential structure determination, a Bayesian approach to integrative modeling of biomolecular complexes with hybrid structural data. I will introduce probabilistic models for cryo-EM maps and outline Markov chain Monte Carlo algorithms for sampling model structures from the posterior distribution. I will focus on rigid and flexible modeling with cryo-EM data and discuss some of the computational challenges of Bayesian inference in the context of biomolecular modeling.

## 1. Introduction

Thanks to groundbreaking advances in experimental techniques it has become possible to study the structure of large biological assemblies at increasingly higher resolution. Traditionally, high-resolution biomolecular structure determination was only possible by X-ray crystallography or nuclear magnetic resonance (NMR) in solution (Berman et al., 2000). The application of NMR and X-ray crystallography to larger systems remained challenging due to the sheer size of the system and/or because it was difficult to find suitable crystallization conditions. More recently, emerging methods such as cryo-electron microscopy (cryo-EM) (Frank, 2002; Orlova and Saibil, 2004; Chiu et al., 2005), crosslinking/mass spectrometry (Gingras et al., 2007; Rappsilber, 2011) and solid-state NMR (Yan et al., 2013) have started to provide exciting insights into the structure of large macromolecular assemblies that was previously very difficult, if not impossible to obtain. In particular, cryo-EM has reached near-atomic and in some cases even atomic resolution over the last 5 years (Bai et al., 2015; Fischer et al., 2015; Khatter et al., 2015). The EM databank (EMDB) (Lawson et al., 2011) stores an increasing number of high-resolution EM reconstructions. Several biologically essential assemblies that resisted high-resolution studies have recently been characterized by cryo-EM including spliceosomal complexes (Yan et al., 2015; Agafonov et al., 2016; Galej et al., 2016; Rauhut et al., 2016; Wan et al., 2016), eukaryotic ribosomes (Anger et al., 2013; Khatter et al., 2015), and transcription initiation complexes (Plaschka et al., 2015).

Although several powerful experimental techniques are available that allow us to study the structure of large biomolecular systems, we need computational methods that assist us in integrative modeling with diverse structural data (Sali et al., 2003; Robinson et al., 2007; Ward et al., 2013). The reasons for developing new computational methods are both of a principled and practical nature.

Structural models built from hybrid data should be as objective as possible and ideally not be biased by a human modeler, therefore automated computational modeling tools are indispensable (Karaca and Bonvin, 2013; Villa and Lasker, 2014; Schröder, 2015). The models should be compatible with all of the available data, which might come from different experimental sources. The modeling software should also be able to integrate data-independent prior information about the system.

Most existing refinement and modeling software focuses on structural data of a particular type. For example, a number of software packages for X-ray structure refinement or modeling with NMR restraints exist. To use these packages for modeling with hybrid data is often difficult and involves some sort of tweaking. We therefore need a versatile software that can integrate diverse types of structural information (Russel et al., 2012).

Every software for integrative modeling with hybrid data has to address the following questions: How much weight should the various pieces of information be given? How to deal with datasets that (partially) contradict some of the other datasets? Obviously, the weights can have a strong impact on the final structure (Brünger, 1992; Habeck et al., 2006), and it would be desirable to choose the weights in a data-driven, self-adaptive fashion. Because the individual datasets themselves typically provide only ambiguous structural information, we have to fit the model against all data simultaneously to obtain the least ambiguous result. What is a good representation of the remaining uncertainty about the structure? We need to represent the ambiguity of the structural model adequately.

The software should also be able to integrate data of varying resolution. A common scenario is that high-resolution information about the subunits in isolation is available (Esquivel-Rodríguez and Kihara, 2013), such that modeling the complex appears to be simple: we just need to put the pieces together. However, even in this seemingly simple situation several issues need to be considered.

The formation of the complex is often accompanied by a conformational change in the subunits (Gerstein et al., 1994). How much should we deviate from the known structures of the free subunits in order to fit the data of the complex? If the data is sparse (e.g., crosslinking or NMR data) or of a medium resolution, there is the risk of overfitting the data.

Another practical problem is the enormous size of the systems that can comprise tens of thousands up to millions of atoms. Is there enough information to determine the position of all atoms? Or should we rather lower our goal and aim for a coarse-grained, intermediate resolution model?

At the source of many of these issues is the question of how to deal with uncertainty in the data and about our model. We need a mathematical framework to quantitatively represent any uncertainty in the process that takes us from the input data to the final model. The framework should allow us to follow the propagation of the uncertainty about a biomolecular structure as we combine data from diverse sources and to compute structural error bars that reflect the degree of uncertainty.

Bayesian probability theory is a unique and objective mathematical framework for quantitative inference from limited, diverse and uncertain information (Cox, 1946; Jaynes, 2003; MacKay, 2003). The essence of the Bayesian approach is that any probability should be interpreted as incomplete information about a quantity rather than a frequency of occurrence. Highly ambiguous and uncertain information results in multi-modal distributions that are spread out over many parameter values. Markov chain Monte Carlo (MCMC) methods (Liu, 2001) allow us to apply the Bayesian formalism in practice even to highly complex data and models.

More than a decade ago, Bayesian methods have been introduced for protein structure determination from solution NMR data (Rieping et al., 2005; Habeck, 2012). In this article, I will describe recent developments in Bayesian integrative modeling with hybrid data.

## 2. Methods

### 2.1. Inferential structure determination

Inferential structure determination (ISD) is the first strictly statistical approach to biomolecular modeling (Habeck et al., 2005a; Rieping et al., 2005). Originally ISD was developed for solution NMR data on small protein domains (Rieping et al., 2008; Habeck, 2012). But the basic principle can be applied to large systems and diverse structural data (Bayrhuber et al., 2008; Shahid et al., 2012; Habenstein et al., 2015).

At the core of the ISD approach is a probabilistic formulation of the structure determination problem. We have to distinguish two principal types of information that guide us in the modeling of a biomolecular structure: the experimental data *D* and data-independent prior information *I* about biomolecular structures. All the information is encoded statistically through conditional probabilities. The probability:

Pr(D|θ,I)

quantifies how probable it is to observe data *D* if the actual configuration of the system is θ. Pr(*D*|θ, *I*) is called the *likelihood* function. The prior probability:

Pr(θ|I)

expresses what we know about reasonable system configurations θ without observing any data.

Probability calculus allows us to combine both types of information and to derive a *posterior* distribution over all conformational degrees of freedom by invoking Bayes' theorem (Jaynes, 2003):

$$\mathrm{Pr}(\theta |D,I)=\frac{1}{\mathrm{Pr}(D|I)}\mathrm{Pr}(D|\theta ,I)\;\mathrm{Pr}(\theta |I).$$

The posterior Pr(θ|*D, I*) expresses what we know about the unknown structure given the experimental data *D* and our prior knowledge *I*. The probability Pr(*D*|*I*) (the so-called model evidence) can be ignored if we are only interested in estimating θ, because Pr(*D*|*I*) does not depend on θ. However, if we aim to compare different prior or modeling assumptions, it will be important to calculate Pr(*D*|*I*) (Habeck, 2011; Mechelke and Habeck, 2012, 2014; Knuth et al., 2015).

Often, we need to introduce additional unknown parameters to express our prior information or to model the experimental data. Let's denote these parameters by ξ; in statistical parlance, ξ are *nuisance parameters*. It is straightforward to infer both θ and ξ from the experimental data. All we need to do is to introduce a prior probability for the model parameters ξ and to invoke Bayes' theorem on the joint parameter space:

$$\mathrm{Pr}(\theta ,\xi |D,I)=\frac{1}{\mathrm{Pr}(D|I)}\mathrm{Pr}(D|\theta ,\xi ,I)\;\mathrm{Pr}(\theta |I)\;\mathrm{Pr}(\xi |I).$$

where we assumed that θ and ξ are independent *a priori*: Pr(θ, ξ|*I*) = Pr(θ|*I*) Pr(ξ|*I*). It is straightforward to relax this assumption if necessary.

The posterior probability Pr(θ, ξ|*D, I*) encodes all available information about the unknown parameters. In biomolecular structure determination, the posterior is typically too complex to do any further analytical calculations. By drawing Monte Carlo samples from Pr(θ, ξ|*D, I*) we generate a finite approximation of the posterior (Liu, 2001). These samples can be used to compute expectations and variances over the unknown parameters and thereby estimate the parameters and compute error bars.

### 2.2. Probabilistic models for hybrid data

Before we can launch an ISD calculation, we need to choose a likelihood Pr(*D*|θ, ξ, *I*) and the priors Pr(θ|*I*) and Pr(ξ|*I*). The application of ISD to multiple datasets *D*_*i*_ is straightforward: Pr(*D*|θ, ξ, *I*) = ∏_*i*_ Pr(*D*_*i*_|θ, ξ). Each dataset is described independently with an appropriate probabilistic model; all datasets are integrated by simply multiplying all factors representing the various datasets. Because probabilities for different datasets are calibrated (they all normalize to one), there is no issue of weighing the different datasets relative to each other.

We use a Boltzmann distribution as a prior over the conformational degrees of freedom:

(1) $$\mathrm{Pr}(\theta |I)=\frac{1}{Z}\exp\{-E(\theta )\}$$

where *E*(θ) is a force field. ISD currently supports two force fields: a quartic repulsion term that lacks any attractive interaction, and a linearly ramped Lennard-Jones potential (see Habeck, 2011; Mechelke and Habeck, 2012 for more details). The prior distribution Pr(θ|*I*) allows us to restrict the conformational degrees of freedom such that reasonable model structures are preferred (for example, structures that are free of atom-atom clashes and have well-packed interfaces). The prior distribution over the model parameters Pr(ξ|*I*) is typically of a standard form and chosen such that sampling with MCMC is straightforward.

#### 2.2.1. Probabilistic model for EM maps

The result of a cryo-EM study is a 3D reconstruction of the structure, which typically comes in the form of a regular cubic grid with equal grid spacing in all three spatial directions. To construct a probabilistic model for 3D reconstructions, we first need a mathematical relation that allows us to compute a theoretical density map from a given structure θ. ISD's current model for density maps is quite simple. The theoretical map is obtained from an atomic model by placing spherical Gaussians of the same size and weight at each atom. The theoretical density at 3D position *x* is:

(2) $$\rho (x;\theta ,\sigma )=\sum_{k}\frac{1}{{\left(2\pi \sigma^{2}\right)}^{3/2}}\exp\{-\frac{1}{2\sigma^{2}}\|x-x_{k}(\theta ){\|}^{2}\}$$

where the index *k* runs over all atoms that contribute to the density and *x*_*k*_(θ) is the 3D position of the *k*-th atom in the structure parameterized by the conformational degrees of freedom θ. The theoretical density map can be interpreted as a blurred version of an atomic map with infinite resolution:

$$\rho (x;\theta ,\sigma )=g_{\sigma }*\rho (x;\theta ,0)\text{ with }\rho (x;\theta ,0)=\sum_{k}\delta [x-x_{k}(\theta )]$$

where δ is the Dirac delta function, *g*_σ_ is a Gaussian blur kernel with bandwidth σ and ^*^ denotes a 3D convolution. Model (2) is admittedly simplistic and valid only for modeling protein complexes at intermediate to low resolutions. For high-resolution maps and/or the modeling of protein/nucleic acid complexes the model should also incorporate atom-wise weights (proportional to atom mass) as well as scattering and temperature factors.

Let us assume that experimental values ρ_*n*_ are available at positions *x*_*n*_ (*n* = 1, …, *N*) which are typically the centers of voxels that make up a cubic grid. The discrepancy between the experimental map ρ_*n*_ and the theoretical map ρ(*x*_*n*_; θ, σ) can be assessed with a Gaussian distribution. Alternative error models for density maps have been proposed (Vasishtan and Topf, 2011), but the Gaussian model is still the most widely used model.

The likelihood function resulting from a Gaussian model is:

(3) $$\begin{array}{l} \mathrm{Pr}(\rho |\theta ,\xi ,I)=\prod_{n=1}^{N}{\left(\frac{\lambda }{2\pi }\right)}^{1/2}\exp\left\{-\frac{\lambda }{2}{\left[\rho_{n}-\alpha \rho (x_{n};\theta ,\sigma )\right]}^{2}\right\}\\ \;={\left(\frac{\lambda }{2\pi }\right)}^{N/2}\exp\{-\frac{\lambda }{2}\sum_{n}{\left[\rho_{n}-\alpha \rho (x_{n};\theta ,\sigma )\right]}^{2}\} \end{array}$$

where the calibration factor α was introduced. There are three nuisance parameters ξ = (σ, α, λ). Typically, the bandwidth of the blur kernel σ is set to a constant value which depends on the resolution of the map. For example, the default value in Chimera (Pettersen et al., 2004) is σ = 0.225 × resolution. For this fixed choice of the bandwidth, σ can be absorbed into the background information *I*. However, it is also possible to estimate σ along with the other nuisance parameters and the conformational degrees of freedom.

To estimate the scaling parameter, we have to look at the conditional posterior distribution:

$$\begin{array}{l} \mathrm{Pr}(\alpha |\lambda ,\theta ,D,I)\propto \mathrm{Pr}(\alpha |I)\times \exp\{-\frac{\lambda \|\rho (\theta ,\sigma ){\|}^{2}}{2}\\ \;{\left(\alpha -\frac{\sum_{n}\rho_{n}\rho (x_{n};\theta ,\sigma )}{\|\rho (\theta ,\sigma ){\|}^{2}}\right)}^{2}\} \end{array}$$

where $||\rho ||=\sqrt{\underset{n}{\sum }\rho_{n}^{2}}$. The second factor is a Gaussian centered about the estimator:

(4) $$\hat{\alpha }(\theta ,\sigma )=\frac{\sum_{n}\rho_{n}\rho (x_{n};\theta ,\sigma )}{\|\rho (\theta ,\sigma ){\|}^{2}}$$

which is the slope of a straight line relating the calculated volume ρ(*x*_*n*_; θ, σ) to the observed density ρ_*n*_.

The Gaussian model is directly related to the cross-correlation coefficient, which is often used to compare EM maps. To see this, let's integrate out the unknown scaling factor α. If we ignore the fact that α should be positive and choose a uniform (improper) prior over α (i.e., Pr(α|*I*) = const), we can analytically integrate out α to obtain a new likelihood that no longer depends on α (this procedure is also called marginalization in Bayesian statistics, Habeck et al., 2005a):

(5) $$\begin{array}{l} \mathrm{Pr}(\rho |\theta ,\lambda ,I)=\int \text{d}\alpha \mathrm{Pr}(\rho |\theta ,\alpha ,\lambda ,I)\;\mathrm{Pr}(\alpha |I)\propto \lambda^{(N-1)/2}\\ \;\exp\{-\frac{\lambda \|\rho {\|}^{2}}{2}[1-C^{2}(\theta )]\} \end{array}$$

where

$$C(\theta )=\frac{\sum_{n}\rho_{n}\rho (x_{n};\theta ,\sigma )}{\left\|\rho \|\|\rho (\theta ,\sigma )\right\|}$$

is the cross-correlation between the experimental and the theoretical map. The effective likelihood function (Equation 5) attains its maximum when the cross-correlation coefficient is one. Whenever we assess the goodness of fit between the model and the experimental map by means of the cross-correlation coefficient, we implicitly assume that the error of the EM map follows a Gaussian distribution.

The parameter λ is the inverse variance of the Gaussian likelihood (Equation 3) and called the *precision* of the model (Bernardo and Smith, 2009). It is also possible to estimate the precision λ of the fit between the experimental and the theoretical density map. The parameter λ assesses how well the experimental and theoretical map agree on average. For large λ, the experimental map is very reliable and imposes a strong force on the model to adapt itself such that the calculated map reproduces the observed map as closely as possible. Assuming Jeffreys's prior for the precision, i.e., Pr(λ|*I*) = 1/λ, the conditional posterior of the precision is a Gamma distribution (Habeck et al., 2006):

(6) $$\mathrm{Pr}(\lambda |\theta ,\alpha ,\rho ,I)\propto \lambda^{N/2-1}\exp\{-\lambda E_{\text{map}}(\theta ,\alpha )\}$$

where the least-squares residual

$$E_{\text{map}}(\theta ,\alpha )=\frac{1}{2}\sum_{n}{\left[\rho_{n}-\alpha \rho (x_{n};\theta ,\sigma )\right]}^{2}$$

is the restraint energy resulting from the Gaussian model of the experimental EM map. The expected value of the precision given the experimental map ρ and all unknown parameters is the inverse mean-squared error:

(7) $$\hat{\lambda }(\theta ,\alpha )\approx \frac{N}{2E_{\text{map}}(\theta ,\alpha )}.$$

Estimator (Equation 7) tells us that the precision of the map increases when the fit between the observed map and the calculated map improves. This seems reasonable, but there is a problem.

Typically, EM maps are surrounded by bordering layers of low density voxels (ρ_*n*_ ≈ 0). If we classify all voxels into *N*_1_ voxels that contain density of the biomolecular assembly and *N*_0_ voxels that carry only noise or zero density, we have *N* = *N*_0_ + *N*_1_. By increasing *N*_0_ (e.g., by zero padding) the goodness of fit *E*_map_ does not change or changes only very little, such that we can artificially increase the apparent precision of the density map simply by increasing *N*_0_:

$$\hat{\lambda }(\theta ,\alpha )\approx \frac{N_{0}+N_{1}}{2E_{\text{map}}(\theta ,\alpha )}\geq \frac{N_{1}}{2E_{\text{map}}(\theta ,\alpha )}.$$

To obtain a realistic estimate of λ, we should only fit those voxels that carry real density.

In principle, the task of classifying voxels into noise and non-noise voxels is an inference problem in itself: we would have to introduce a mask that tells us whether a voxel carries true signal or not. For the sake of simplicity we do not introduce an adaptive mask that we estimate along with with the model parameters, but restrict the fitting to voxels that are likely to carry the true signal. These voxels are identified in a couple of preparatory steps, which I will outline in the next section.

If we look at the conditional posterior of the conformational degrees of freedom θ, we find that:

(8) $$\mathrm{Pr}(\theta |\xi ,\rho ,I)\propto \exp\{-E(\theta )-\lambda E_{\text{map}}(\theta ,\alpha )\}.$$

By taking the negative logarithm of the posterior probability, we obtain a hybrid energy function (Jack and Levitt, 1978; Brünger and Nilges, 1993; Habeck et al., 2005a):

(9) $$E_{\text{hybrid}}(\theta )=E(\theta )+\lambda E_{\text{map}}(\theta ,\alpha ).$$

The precision acts as a weighting factor for the EM map (Habeck et al., 2006). If λ is too large, the forces from the EM term can bias the final structure (overfitting). Therefore, it is important to obtain a realistic estimate of λ.

#### 2.2.2. Preparation of EM maps

ISD carries out several preparatory steps before modeling with EM maps starts: thresholding, cropping, decimation, and masking. These steps improve the speed of fitting and are necessary to obtain a meaningful estimate of the precision of the density map.

Typically the user provides a threshold ρ_min_ above which the density shows the particle. ISD clips the density at ρ_min_, i.e., all values greater than the threshold are set to the threshold. After clipping, the density is shifted by subtracting the threshold such that the smallest experimental density is zero:

(10) $$\rho_{n}\leftarrow \{\begin{array}{ccc} \rho_{n}-\rho_{\min} & ; & \rho_{n}\geq \rho_{\min}\\ 0 & ; & \rho_{n}<\rho_{\min} \end{array}$$

After thresholding all ρ_*n*_ ≥ 0. To reduce the map to those voxels that carry the real signal, a cropping operation is applied to reduce the 3D grid to a minimum size. Cropping removes bordering layers which only contain zero-density voxels analogous to an auto crop in image processing programs.

To represent the assumption that the structure is entirely covered by the thresholded density map, ISD introduces a box prior, which confines the system to lie inside the interior of a cubic box that coincides with the boundary of the 3D map. The box is parameterized by its lower left and upper right corner where the lower left corner is located at the origin of the 3D grid on which the thresholded EM map is evaluated. The box has a soft boundary which is implemented as a logistic function with finite steepness γ:

(11) $$s_{\gamma }(x)=\frac{1}{1+e^{-\gamma x}}$$

where typically γ = 1Åmplete prior over the conformational degre^−1^. The complete prior over the conformational degrees of freedom is:

(12) $$\mathrm{Pr}(\theta |I)\propto \exp\{-E(\theta )\}\prod_{k}\prod_{d=1}^{3}s_{\gamma }(x_{kd}(\theta )-l_{d})s_{\gamma }(u_{d}-x_{kd}(\theta ))$$

where *l*_*d*_, *u*_*d*_ are the spatial coordinates of the lower left / upper right corner of the bounding box of the EM map and *x*_*kd*_(θ) are the spatial coordinates of the *k*-th atom.

The Gaussian likelihood (Equation 3) is only valid for voxels that carry signal. Let us introduce a binary mask *m*_*n*_ ∈ {0, 1} which indicates for each voxel, if it carries signal (*m*_*n*_ = 1) or noise (*m*_*n*_ = 0). The modified Gaussian likelihood is:

(13) $$\begin{array}{l} \mathrm{Pr}(\rho |\theta ,\xi ,I)={\left(\frac{\lambda }{2\pi }\right)}^{\sum_{n}m_{n}/2}\\ \;\exp\{-\frac{\lambda }{2}{\sum_{n}m_{n}[\rho_{n}-\alpha \rho (x_{n};\theta ,\sigma )]}^{2}\}. \end{array}$$

As mentioned above, the mask *m*_*n*_ should in principle be also considered an unknown parameter and therefore be estimated along with the other unknown quantities. However, this is currently not implemented in ISD and therefore *m* is part of the background information *I*.

Another parameter that we have to consider is the spacing of the EM map. The Gaussian likelihood assumes that the discrepancy between the experimental and calculated map is independent from voxel to voxel and shows no spatial correlations. However, this assumption is violated when the size of the voxels becomes too small. By resampling the experimental map on a finer grid, we could artificially increase the number of data points, which would result in an increase of the estimated weight λ. Therefore, EM maps are typically downsampled in ISD such that the spacing is roughly 2 × σ. A more rigorous treatment that accounts for spatial correlations between neighboring voxels is currently under development.

#### 2.2.3. Conformational degrees of freedom

ISD supports multiple parameterizations for biomolecular systems. ISD typically decouples internal degrees of freedom from rigid external degrees of freedom, although modeling based on Cartesian coordinates is also supported. In case we want to model the internal flexibility of the subunits of a biomolecular assembly, ISD uses dihedral angles to parameterize the atom positions. The external degrees of freedom are three translational and three rotational degrees of freedom. To parameterize the rotation matrices, ISD uses a Lie group representation (Gallego and Yezzi, 2015). It is also possible to model symmetric assemblies by using virtual copies of the symmetry mates. ISD supports cyclic, dihedral and helical symmetry. The parameters of a helical symmetry can be estimated along with the conformational degrees of freedom.

To sample the conformational degrees of freedom θ, ISD uses the gradient of the log posterior probability (i.e., the gradient of the hybrid energy). Typically it is straightforward to compute the gradient with respect to the Cartesian coordinates. The Cartesian gradient is mapped onto the conformational degrees of freedom by virtue of the chain rule. This requires us to evaluate the Jacobian of the parameterization. In case of dihedral angles, there is an efficient recursive algorithm that avoids building up the full Jacobian matrix by traversing the tree of covalent bonds.

### 2.3. Markov Chain Monte Carlo for biomolecular modeling

The posterior probability Pr(θ, ξ|*D, I*) encodes everything that can be said about the conformational degrees of freedom θ and the nuisance parameters ξ in the light of the experimental data *D* and our modeling assumptions *I*. Because Pr(θ, ξ|*D, I*) is a high-dimensional probability distribution that is not suited for analytical computations, we explore Pr(θ, ξ|*D, I*) by drawing random samples from it. Sampling from Pr(θ, ξ|*D, I*) is based on Markov chain Monte Carlo (MCMC) (Liu, 2001). An MCMC algorithm simulates a Markov chain over (θ, ξ) space whose stationary distribution is the posterior Pr(θ, ξ|*D, I*). After convergence of the Markov chain, the generated θ, ξ are valid samples from Pr(θ, ξ|*D, I*). The samples can be used to compute expected values, variances and other statistics that characterize the posterior distribution. If we were to construct a multi-dimensional histogram from the θ, ξ samples, it would approximate the posterior distribution. The longer we run the Markov chain, the closer we get to the posterior distribution.

#### 2.3.1. Gibbs sampling

Gibbs sampling (Geman and Geman, 1984) is an iterative MCMC algorithm that decomposes sampling from Pr(θ, ξ|*D, I*) into two successive steps, which are repeated:

(14) $$\begin{array}{l} \theta^{\left(t+1\right)}\sim \mathrm{Pr}(\theta |\xi^{\left(t\right)},D,I)\\ \xi^{\left(t+1\right)}\sim \mathrm{Pr}(\xi |\theta^{\left(t+1\right)},D,I) \end{array}$$

where *t* is an iteration index (pseudo time) and the superindex (*t*) marks samples generated in the *t*-th iteration; the notation ~ means “sampled from.” It can be shown that the Gibbs sampler (Equation 14) generates valid samples from the joint distribution Pr(θ, ξ|*D, I*).

To implement a Gibbs sampler, we need to compute the conditional posterior distributions Pr(θ|ξ, *D, I*) and Pr(ξ|θ, *D, I*). The conditional posterior over the conformational degrees of freedom involves the hybrid energy (Equation 9):

(15) $$\mathrm{Pr}(\theta |\xi ,D,I)\propto \exp\{-\lambda E_{\text{map}}(\theta ,\alpha )-E(\theta )\}.$$

Sampling of the nuisance parameters is most easily done by applying a Gibbs sampling strategy to Pr(ξ|θ, *D, I*) itself. We break down the second step in scheme (14) into the generation of α and λ samples according to:

(16) $$\begin{array}{l} \alpha^{\left(t+1\right)}\sim \mathrm{Pr}(\alpha |\lambda^{\left(t\right)},\theta^{\left(t+1\right)},D,I)\\ \lambda^{\left(t+1\right)}\sim \mathrm{Pr}(\lambda |\alpha^{\left(t+1\right)},\theta^{\left(t+1\right)},D,I) \end{array}$$

The conditional posteriors for the individual nuisance parameters, e.g., Pr(λ|α, θ, *D, I*), have been discussed in the previous section. Often these distributions are of a standard form and can be sampled directly using random number generators. For example, the conditional posterior of the precision λ is a Gamma distribution (Equation 6). Efficient algorithms for generating variates from a Gamma distribution exist (Devroye, 1986).

#### 2.3.2. Hamiltonian Monte Carlo

Sampling the conformational degrees of freedom θ from the conditional posterior (Equation 9) is the most challenging step in an ISD calculation. Typically, the conformational degrees of freedom are highly coupled, and Pr(θ|ξ, *D, I*) exhibits multiple peaks. A powerful variant of Metropolis Monte Carlo (Metropolis et al., 1957) is the Hybrid Monte Carlo method, also known as Hamiltonian Monte Carlo (HMC) (Duane et al., 1987; Neal, 2010). The improvement over the simple Metropolis sampler is achieved by using a more efficient proposal step. In the standard version of Metroplis Monte Carlo, new candidate structures are proposed by randomly perturbing a conformational degree of freedom. The perturbation is either accepted or rejected depending on whether it produced an acceptable change in the hybrid energy or not. This kind of proposal results in a random walk in conformational space, which explores the space very inefficiently, because typically we can only apply small perturbations to the structure without increasing the hybrid energy by an unacceptable amount.

HMC proposes the candidate structure by running a short molecular dynamics trajectory where the hybrid energy plays the role of a force field. This has the advantage that the moves in structure space are adapted to the shape of the posterior distribution and that the conformational degrees of freedom change conjointly rather than one by one. HMC is several orders of magnitude more efficient than random walk Metropolis Monte Carlo, but comes at an additional computational cost. To run the proposal trajectory, one needs to calculate the gradient of the hybrid energy with respect to the conformational degrees of freedom. Since ISD uses non-Cartesian parameterizations, the gradient can be quite involved. Thanks to the chain rule we can break the computation of the gradient into two steps: First, the Cartesian gradient is calculated. In a second step, the Cartesian gradient is projected into the space of the conformational degrees of freedom. ISD implements this projection for dihedral angles and the rotational degrees of freedom of a rigid-body transformation.

#### 2.3.3. Replica-exchange simulation

The posterior distribution arising in an application of ISD, is quite complex and typically shows multiple modes. As we will see in Section 3.3, the posterior distribution encountered in integrative modeling with cryo-EM data is often sharply peaked and exhibits isolated peaks. It is highly challenging to draw conformational samples from such a posterior distribution. ISD uses replica-exchange simulations (also known as parallel tempering) (Swendsen and Wang, 1986; Geyer, 1991) to address the sampling problem.

There are two factors that contribute to the posterior, the prior and the likelihood, and both are difficult to simulate in their own right. Therefore, ISD controls the complexity of each factor independently by introducing two “temperatures” (Habeck et al., 2005b). The first parameter, the inverse temperature β∈[0, 1], scales the likelihood:

$${\left[\mathrm{Pr}(D|\theta ,\xi ,I)\right]}^{\beta };$$

for β = 1 we obviously recover the original likelihood, for β = 0 we completely switch off the data.

The second parameter controls the shape of the conformational prior. Because the non-bonded interactions *E*(θ) span many orders of magnitude, it is highly inefficient to work with the standard Boltzmann ensemble which scales down the non-bonded energy when the temperature is increased. Instead of the Boltzmann ensemble, ISD uses the Tsallis ensemble to smooth out non-bonded interaction (Habeck et al., 2005b) and simulates:

$${\left[1+(q-1)(E(\theta )-E_{\min})\right]}^{-q/(q-1)}$$

where *q* ≥ 1 is the so-called Tsallis *q* and *E*_min_ has to be chosen such that *E*(θ) > *E*_min_ for all structures. For *q* = 1, we recover the standard Boltzmann prior (Equation 1).

The choice of the tempering schedule (i.e., the sequence of β and *q*) is difficult and crucial. We have to trade-off efficiency vs. ergodicity of sampling. With increasing number of temperatures, the overlap between the replicas increases which results in an elevated swapping rate. But with increasing number of replicas the time for round trips increases quadratically, because states diffuse across different temperatures (i.e., there is no directed exchange of states that would aim for rapid mixing of states across different temperatures) (Earl and Deem, 2005). Therefore, we would rather choose a minimal number of replicas such that the smallest swapping rate is maintained.

## 3. Results

In this section, I will illustrate Bayesian integrative modeling with hybrid data focusing on EM maps.

### 3.1. Flexible fitting with Hamiltonian Monte Carlo

ISD can fit known structures and structural models into EM maps. In flexible fitting, we are trying to change the internal structure of a biomolecule so as to better fit an experimental EM map. A couple of software packages for flexible fitting has been published. Normal mode and elastic network methods (Delarue and Dumas, 2004; Tama et al., 2004; Hinsen et al., 2005; Schröder et al., 2007; Jolley et al., 2008; Tan et al., 2008) boost transitions along the principal directions of structural change. Molecular dynamics (MD) based methods (Orzechowski and Tama, 2008; Trabuco et al., 2008) combine a density fitting score with a full-fledged force field. Real-space refinement in Cartesian and internal coordinates, originally developed for X-ray crystallographic data, has been adapted to cryo-EM maps (Fabiola and Chapman, 2005). Rigid-body modeling with Flex-EM (Topf et al., 2008) freezes secondary structure elements and keeps just the linker regions flexible. Fragment-based structure prediction methods such as Rosetta has been combined with density map refinement (DiMaio et al., 2009).

ISD uses dihedral angles to parameterize the structures of the subunits of a macromolecular complex. In addition to the dihedral angles, each subunit has six external degrees of freedom that describe a rigid transformation of the subunit (three translational and three rotational degrees of freedom). The complete list of dihedral angles as well as the translational and rotational degrees of freedom from all subunits makes up the conformational degrees of freedom θ.

To study flexible fitting with ISD, let us first look at a specific example. Adenylate kinase (AK) is a widely used test system to predict and simulate conformational changes in proteins (see e.g., Orzechowski and Tama, 2008; Beckstein et al., 2009; Whitford et al., 2009). AK adopts two conformational states: an open state in which no ligands are bound and a closed state. The overall difference between both states is an RMSD of ~7 Å. The conformational change can be understood as a rigid-body movement of three domains relative to each other: CORE, LID, and NMP-bind. During the conformational change, these three domains maintain their internal structure (Müller et al., 1996; Whitford et al., 2009).

**Figure 1:**
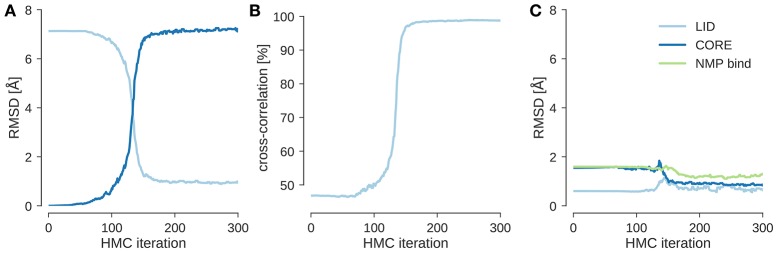
**Flexible fitting of adenylate kinase into a 10 Å map. (A)** Evolution of the RMSD to the initial structure (4ake) shown in dark blue and the target structure (1ake) shown in light blue. **(B)** Evolution of the cross-correlation coefficient during flexible fitting. **(C)** RMSD reduced to Cα atoms that are part of the same rigid domain.

I ran local posterior sampling with HMC starting from the open state (PDB code 4ake) and fitted it into a simulated EM map of the closed state (PDB code 1ake) at 10 Å resolution. Figure 1A shows the evolution of the RMSD to the initial and target structures during flexible fitting. The simulation starts at an RMSD of about 7 Å and rapidly improves it by optimizing the agreement with the experimental and theoretical maps. This is reflected by the evolution of the cross-correlation coefficient (see Figure 1B), which increases as the RMSD to the target structure decreases. After less than 200 steps of HMC sampling the fitted structure has an RMSD < 1 Å to the target structure and a cross-correlation of almost 100%. During flexible fitting, the structure of the three domains remains intact. This is reflected by the fact that the RMSD restricted to those Cα atoms that belong to the same domain changes only little compared to the change in the overall RMSD (see Figure 1C). Thus, the HMC sampler preserves the integrity of the input structure and introduces larger scale changes only in a few hinge regions.

### 3.2. Flexible fitting benchmark

To systematically validate local flexible fitting of EM maps with ISD, I applied HMC sampling of the posterior distribution to a benchmark proposed by Topf et al. (2008) to test their Flex-EM method. The Flex-EM benchmark comprises various medium sized proteins and simulated EM maps at different resolutions ranging from 4 to 14 Å. For each flexible fitting task of the single-domain subset, I launched an HMC sampler starting from the initial structure as provided by the benchmark. The initial structure was obtained by homology modeling based on a template structure that shows an alternative conformational state. The task is to deform the homology model such that it better agrees with a simulated EM map showing a different conformational state.

**Figure 2:**
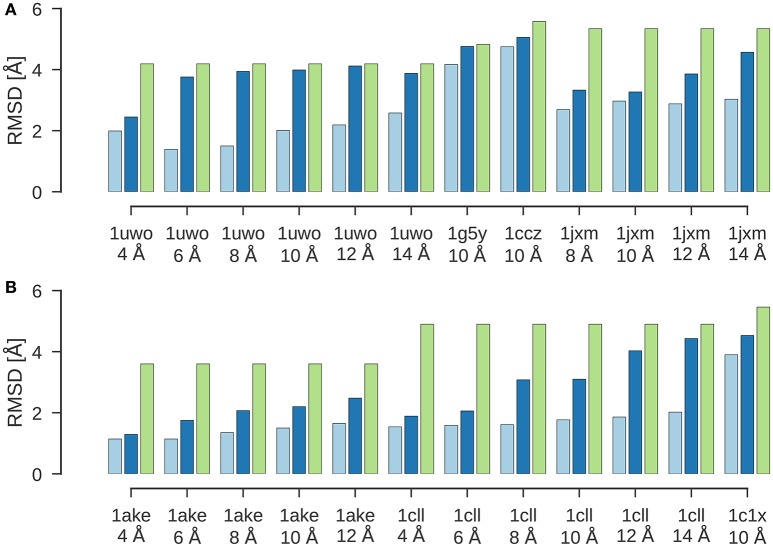
**Flexible fitting benchmark**. Shown are the RMSD values for the final results of flexible fitting with ISD (light blue) and Flex-EM (dark blue) in comparison to the RMSD of the initial structure to the target structure (green). **(A)** Flexible fitting results for 1uwo, 1g5y, 1ccz, 1jxm. **(B)** Flexible fitting results for 1ake, 1cll, 1c1x.

Figure 2 shows the results of a flexible fitting benchmark from Topf et al. (2008). In all cases, ISD improves the fit of the initial structure quite significantly and achieves cross-correlation coefficients above 95%. Moreover, the RMSDs of the final structures fitted with ISD are systematically better than the fits obtained with Flex-EM.

Although flexible fitting with HMC performs well in practice, there are still conceptual problems with this approach. Sampling with HMC does not explore the full posterior distribution, but stays in the vicinity of the initial structure. A truly Bayesian approach, however, aims to explore the entire posterior distribution by using, for example, a full-blown replica simulation. However, global sampling of the posterior will result in many alternative fits of the EM map that will show a large RMSD to the target structure, because the force fields implemented in ISD cannot distinguish between the target structure and other globular structures that fit the density map. A remedy is to not only use the known structure that is fitted against the EM map as the initial structure, but also to develop a probabilistic model that allows for deformations of the known structure. Such a model is currently under development.

### 3.3. Global fitting of symmetric assemblies

Global sampling of the posterior distribution is currently only possible in ISD, if the internal structure of the subunits is kept fixed. The only degrees of freedom are the six external degrees of freedom parameterizing a global rotation and translation of each subunit. The sampling problem arising in global fitting of EM maps is quite severe. To see this, let us first study sampling from the prior (Equation 12), which is the Boltzmann ensemble confined by a soft box containing the experimental density map. Sampling from this prior is a sort of toy version of the density fitting problem. Instead of fitting the assembly against the density map, our aim is to generate non-clashing configurations that lie inside a box which contains the thresholded map. This is an instance of a 3D packing problem, which is NP-hard.

Let us look at a specific example: The symmetric chaperonin GroEL has been studied extensively by cryo-EM, X-ray crystallography and NMR. A 3D reconstruction of GroEL at a resolution of 4.1 Å is available (EMD-6422). The original map spans 240^3^ voxels. The EMDB entry suggests a user-defined threshold of ρ_min_ = 3.5 for visualizing the map. After thresholding (Equation 10) and cropping, the grid has 135 × 133 × 133 voxels, i.e., only ~ 17% of the original volume carries information that is useful for structural modeling. The 3D cropping operation results in a box that spans a volume of 144.5 × 142.3 × 142.3 Å^3^. This example illustrates that thresholding and cropping can achieve a drastic reduction in the number of grid points that have to be evaluated during density fitting.

GroEL exhibits a seven-fold tetrahedral symmetry (D7). Therefore, our task is to sample configurations of the 14-mer that fit inside the box and minimize the overlap between atoms from different subunits. I used a Tsallis replica simulation to sample structures of the GroEL 14-mer. There are only six conformational degrees of freedom: three rotational and three translational degrees of freedom, which determine the position and orientation of a single GroEL subunit. The positions and orientations of the other 13 subunits are generated by the action of the D7 symmetry operator.

Although this is a low-dimensional sampling problem, it turns out to be surprisingly hard. I needed 59 replicas in the Tsallis ensemble to achieve an average swap rate of 38%. If the non-bonded interactions are fully switched on, there are only few arrangements that fit into the box without producing significant clashes between atoms from different subunits. As a consequence, the box prior exhibits a few isolated peaks. The shape of the prior distribution is reminiscent of a golf-course energy landscape and quite different from the funnel-shaped energy landscape imposed by distance restraints.

**Figure 3:**
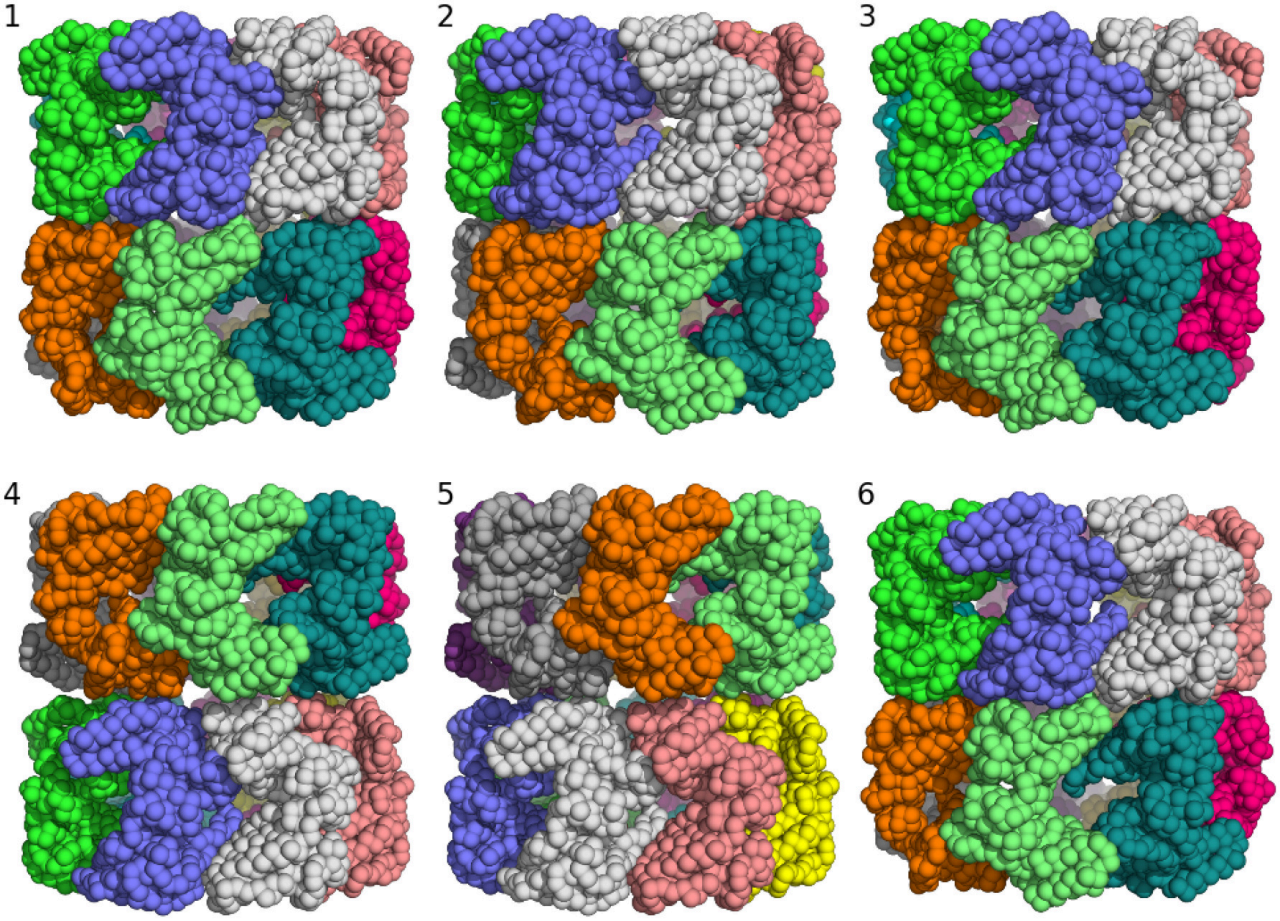
**Major structural clusters of the GroEL 14-mer generated from the prior distribution confined to a box**. Subunits are color coded. The lowest energy clusters are shown on top (structures 1–3). The second lowest energy structures are clusters 4 and 5. Structure 6 is a rare high energy configuration that is also generated by replica-exchange Monte Carlo.

Clustering of the sampled rigid-body degrees of freedom yields six groups of symmetric assemblies that fit into the box (see Figure 3 and Table 1). Each group is defined very precisely with an ensemble RMSD ranging between 0.13 and 0.23 Å over the entire 14-mer. The tightness of the clusters shows that there is only a discrete set of arrangements that fits into the box. The first three clusters achieve the lowest non-bonded energies *E*(θ). The energy of the next two clusters is elevated by 70 units. Replica-exchange Monte Carlo occasionally also samples a high-energy structure (cluster 6). The first five clusters show the same arrangement of the seven-membered ring formed by chains A–G. The RMSD of these chains to the arrangement in the crystal structure is below 0.8 Å; only the last cluster shows a higher RMSD of 4.7 Å. The major difference between the clusters is in how the rings are arranged relative to each other. In clusters 1, 2, 3, and 6, the two rings are oriented in the same fashion as in the crystal structure (with the termini facing each other), whereas clusters 4 and 5 show an inverted orientation.

**Table 1:** **Summary of a clustering analysis of the prior ensemble of GroEL**.

**Cluster**	**av. energy**	**Population [%]**	**Ensemble RMSD**	**RMSD (7-mer) [Å]**	**RMSD (14-mer) [Å]**
1	228.8	22.8	0.2	0.8	7.8
2	234.0	23.1	0.2	0.7	9.0
3	234.1	23.1	0.1	0.7	13.4
4	301.7	19.3	0.2	0.8	71.5
5	301.7	11.5	0.2	0.8	80.2
6	995.5	0.2	0.1	4.7	8.6

*Six major clusters have been identified. Listed are their average non-bonded energy, the RMSD to the average structure within each cluster (precision) and the RMSD (accuracy) to the crystal structure (PDB code 1oel) for a single ring (chains A–G) and the entire 14-mer (chains A–N)*.

Posteriors based on distance data such as those arising in NMR applications exhibit a continuum of high-probability structures. The Markov chain is guided to the most likely structures by a funnel-shaped probability landscape. The distributions arising in EM fitting problems show a very different landscape with multiple isolated peaks that carry similar probability mass and therefore all contribute significantly to the posterior. Rigid-body modeling with EM maps can be viewed as a 3D packing problem. In case of GroEL, the packing constraint from the prior box and the D7 symmetry already determine the overall structure of the assembly to a large degree without any use of the density map. But the tests also show that even sampling from the prior alone can be quite challenging.

The minimum energy assembly sampled from the prior fits the density map only poorly with a cross-correlation of ~10%. Refining the assembly in the presence of the map improves the cross-correlation to 55% and decreases the RMSD of the entire 14-mer to 1.1 Å.

### 3.4. Multi-body modeling of GroEL/ES

In general rigid-body modeling applications, we have to fit multiple rigid bodies into an EM map. I will use the GroEL/ES complex to illustrate multi-body fitting with ISD. GroEL/ES is formed by GroEL and the cochaperonin GroES. GroES interacts with one of the seven-membered rings formed by GroEL after a conformational change has been induced in the subunits. Therefore, the structures of the two GroEL 7-mers are no longer identical, and we have to fit three rigid bodies: one subunit of free GroEL (PDB code 1aon, chain A), one subunit of GroEL in complex with GroES (1aon, chain H), and one subunit of GroES (1aon, chain O). Each of the three subunits is duplicated by the action of a 7-fold cyclic symmetry. The symmetry mates are not represented explicitly, but generated from each of the three rigid bodies. Forces that act on the symmetry mates are backprojected onto the subunit. Therefore, we have a total of 18 conformational degrees of freedom.

**Figure 4:**
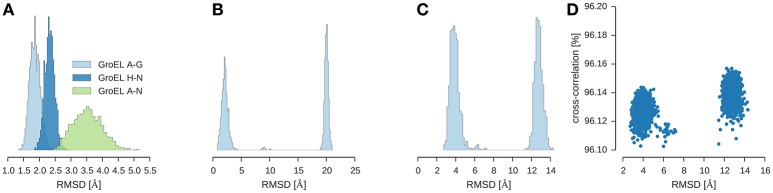
**Multi-body modeling of GroEL/ES**. Shown is the RMSD between structural models obtained by posterior sampling with ISD and the crystal structure (PDB code 1aon). **(A)** RMSD for GroEL subunits for both 7-membered rings (chains A–G and chains H–N) and for the entire 14-mer (chains A–N). **(B)** RMSD for GroES (chains O–U) **(C)** RMSD for the entire 21-mer. **(D)** Correlation between the overall RMSD (21-mer) and the cross-correlation coefficient.

I used ISD to fit GroEL/ES into a 23.5 Å map (Ranson et al., 2001) (EMD-1046). To shortcut the convergence of posterior sampling, I first ran a replica simulation with a Cα representation of the subunits and switched off the non-bonded interactions. With this strategy, the sampler rapidly generates models that achieve a cross-correlation of 96% (see Figure 4D). Inspection of the structures shows that there are two clusters which differ only in the structure of the GroES subunit. The structure of the two GroEL rings is already very close to the crystal structure (1aon) with an RMSD of 3.5 ± 0.5 Å over the 14-mer formed by the GroEL subunits (Figure 4A). The GroES 7-mer arranges in two versions of the ring: One is the correct structure with an RMSD of 2.1 ± 0.6 Å to the crystal structure. The second structure is incorrect with an RMSD of 20.0 ± 0.3 Å. Both structures are almost equally populated. The correct structure is adopted by 51.3% of the structures; the population of the incorrect assembly is 47.7% (see Figure 4B). There is a tiny fraction with a population of ~1% that shows a third arrangement of the GroES subunit (RMSD 9.17 ± 0.51 Å). Figure 4C shows the distribution of the RMSD over the entire assembly.

In a refinement step, I used a full-atom representation of the subunits and switched on the non-bonded energy terms. The RMSD to the crystal structure drops to 1.4 Å without compromising the fit to the EM map: the cross-correlation coefficient of the full-atom structure is still 96%.

### 3.5. Estimation of the precision of an EM map

As outlined in Section 2.2.1, it is challenging to obtain a good estimate of the precision of an EM map, because an EM map typically contains many zero-density voxels in addition to the non-noise voxels, but only voxels carrying a real signal should contribute to the precision. To identify which voxels carry true signal, we would have to first solve the fitting problem. Therefore, both problems, the estimation of a well-fitting structure and the construction of a good mask, are highly related. Moreover, the errors (i.e., the discrepancy between the experimental and calculated maps) are spatially correlated, but the Gaussian model (3) treats them as completely independent observations, which also results in an artificial increase in the precision. The reason for the latter effect is the following: If errors are correlated, the effective number of data points is smaller than the number of voxels (Sivia, 2004). According to Equation (7) the precision of the map is proportional to the number of voxels for the simple Gaussian model, the precision will therefore be overestimated, if the errors between neighboring voxels are correlated.

**Figure 5:**
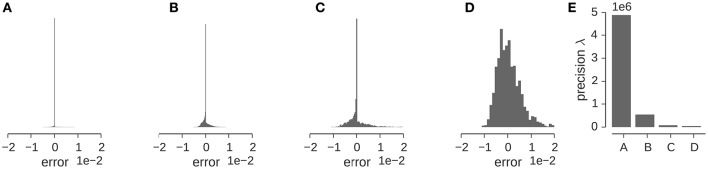
**Estimation of the precision λ of the GroEL/ES map. (A–D)** Show the distribution of the “error” (or discrepancy) between the experimental and calculated maps ρ_*n*_ − ρ(*x*_*n*_; θ, σ). Error distribution for the full map **(A)**, full map after cropping **(B)**, the downsampled and cropped map **(C)**, the downsampled, cropped and masked map **(D)**. **(E)** Estimated precision for the different input maps used in multi-body fitting.

Let us illustrate the various factors that influence the precision for a concrete example. Figure 5 shows the distribution of the discrepancy between the experimental and the calculated density map for the GroEL/ES map analyzed in the previous section. The Gaussian likelihood assumes that this distribution has a bell-shaped curve whose width is determined by the precision λ. The distribution of the discrepancy ϵ_*n*_ = ρ_*n*_ − ρ(*x*_*n*_; θ, σ) is shown in (Figures 5A–D) for various stages of preprocessing. The original map contains many low-density voxels that lead to a very sharp, dominating peak at zero in the distribution of ϵ_*n*_ (Figure 5A). Cropping (Figure 5B) and subsequent decimation (Figure 5C) chops away many of the zero-density voxels and decreases the detrimental effect of the low-density voxels. However, the distribution of ϵ_*n*_ is only captured well by a Gaussian, if we mask out low-density voxels (see Figure 5D). The effect of the preprocessing steps on the estimated precision is shown in Figure 5E. Each of the preparation steps lowers the estimated precision by orders of magnitude.

## 4. Conclusion

This article discusses how ISD incorporates EM maps into a structure calculation and demonstrates some aspects of Bayesian integrative modeling with EM data. The Bayesian framework is highly suited to address issues in structural modeling with hybrid data such as how to weigh multiple datasets relative to each other. The major bottleneck of an inferential structure determination is conformational sampling. The posterior distribution arising in EM fitting poses a challenging sampling problem, which can be overcome with replica-exchange Monte Carlo.

The article does not cover crosslinking/mass spectrometry and solid-state NMR, which are complementary methods for characterizing the structure of large assemblies. ISD has also been used to model biomolecular assemblies from solid-state NMR data. For example, we have used ISD to compute the structure of the membrane domain of the trimeric autotransporter adhesin YadA (Shahid et al., 2012). We modeled a fully flexible subunit in the presence of a cyclic trimer symmetry. Although the data are highly ambiguous due to the imprecision of solid-state NMR restraints and the trimer symmetry, ISD was able to determine the correct structure of the YadA membrane anchor domain. Another example is our recent structure of a type 1 pilus FimA from *E. coli* (Habenstein et al., 2015). Here solid-state NMR and scanning electron microscopy data were combined with solution NMR data to estimate the internal structure of the subunit as well as the parameters of the helical symmetry of the FimA pilus. Also modeling with crosslinking data is possible with ISD, e.g., Carstens et al. (2016) discuss chromosome structure modeling. However, the use of crosslinking data for modeling macromolecular complexes still needs to be benchmarked thoroughly. A common scenario is to combine cryo-EM with crosslinking data, which also needs to be tested systematically with ISD. A Bayesian approach to modeling macromolecular assemblies with crosslinking data has been proposed recently by Ferber et al. (2016).

Future work will focus on various aspects of modeling with hybrid data. One goal is to develop a better model for EM maps that incorporates the various preprocessing steps discussed in Section 2.2.2. The model will incorporate a mask that will be estimated along with the other unknown parameters. Moreover, we will develop a likelihood function that accounts for spatial correlations between errors in the density map. Another goal is to support modeling with coarse-grained representations of biomolecular systems (Tozzini, 2005; Saunders and Voth, 2013). Especially, for very large systems it will be critical to work with a multiscale representation to enable exhaustive conformational sampling. We are already using highly coarse-grained models for modeling the 3D structure of chromosomes and genomes from chromosome conformation capture data (Carstens et al., 2016).

## Author contributions

MH designed and performed research and wrote the manuscript.

## Funding

The author acknowledges funding from the German Research Foundation (DFG) (SFB 860, Project B09).

### Conflict of interest statement

The author declares that the research was conducted in the absence of any commercial or financial relationships that could be construed as a potential conflict of interest.
